# Supplementary material for: Translational Insights and New Therapeutic Perspectives in Head and Neck Tumors
Source: Biomedicines. 2021 Aug 19;9(8):1045. doi: 10.3390/biomedicines9081045 (PMC8391435; doi:10.3390/biomedicines9081045)
Supplement: Supplementary file 1 [file biomedicines-09-01045-s001.zip › biomedicines-1312413-supplementary.pdf]

Methods for literature search.

We conducted a comprehensive, critical, and objective analysis of the current knowledge on a topic. Although a systematic strategy was not followed, a literature search was carried out in the main databases (PubMed, EMBASE, SCOPUS, and Cochrane Library). The following words were used for the research: “squamous cell carcinoma of the head and neck”, “translational research”, “intracellular pathways”, “targeted therapy”, “immunotherapy”, “oncogenes”, and “tumor suppressor genes”.
